# Fast, approximation-free molecular simulation of the SPC/Fw water model using non-reversible Markov chains

**DOI:** 10.1038/s41598-024-66172-0

**Published:** 2024-07-16

**Authors:** Philipp Höllmer, A. C. Maggs, Werner Krauth

**Affiliations:** 1 Physikalisches Institut and Bethe Center for Theoretical Physics, University of Bonn, Nussallee 12, 53115 Bonn, Germany; 2 CNRS UMR7083, ESPCI Paris, Université PSL, 10 Rue Vauquelin, 75005 Paris, France; 3 Laboratoire de Physique de l’Ecole normale supérieure, ENS, Université PSL, CNRS, Sorbonne Université, Université de Paris-Cité, Paris, France; 4 Rudolf Peierls Centre for Theoretical Physics, University of Oxford, Oxford, UK

**Keywords:** Molecular simulation, Non-reversible Markov chains, Monte Carlo methods, Long-range interactions, Statistical physics, thermodynamics and nonlinear dynamics, Physics, Chemical physics

## Abstract

In a world made of atoms, computer simulations of molecular systems such as proteins in water play an enormous role in science. Software packages for molecular simulation have been developed for decades. They all discretize Hamilton’s equations of motion and treat long-range potentials through cutoffs or discretization of reciprocal space. This introduces severe approximations and artifacts that must be controlled algorithmically. Here, we bring to fruition a paradigm for molecular simulation that relies on modern concepts in statistics to explore the thermodynamic equilibrium with an exact and efficient non-reversible Markov process. It is free of all discretizations, approximations, and cutoffs. We explicitly demonstrate that this approach reaches a break-even point with traditional molecular simulation performed at high precision, but without any of its approximations. We stress the potential of our paradigm for crucial applications in biophysics and other fields, and as a practical approach to molecular simulation. We set out a strategy to reach our goal of rigorous molecular simulation.

The fact that all matter consists of atoms was described by R. P. Feynman as the greatest insight of science^1^. The consequence that matter can be modeled on a computer by following the motion of its atoms leads to the founding paradigm of molecular simulation. It tracks the dynamics and explores the thermodynamic equilibrium of complex molecular systems, for example, a peptide in an explicit water solution with tens of thousands of atoms, all interacting through classical empirical potentials^2^. Molecular simulation is of enormous importance to numerous fields ranging from biology and physics to engineering^3,4^. Powerful computer packages have been developed over decades. They all implement the molecular-dynamics approach^5–9^, that is, compute the forces on all atoms at discretized time steps and then update the atomic positions and velocities to integrate Hamilton’s equations of motion. This approach may yield static and dynamic properties both in non-equilibrium and in thermodynamic equilibrium.

A voluminous literature is dedicated to the analysis and control of time-discretization errors in molecular-dynamics simulations (see, e.g., Ref.^10^). Thermostats, understood as “necessary evils”^11^, mimic the effect of a coupled thermal reservoir and, in a symptomatic but non-curative treatment, hide the accumulated errors. The limiting factor in molecular dynamics is the computation of forces. The Lennard-Jones interaction is typically cut off beyond a certain distance so that only a few neighbors exert their force on any given atom. This cutoff (or a discretization of reciprocal space akin to the approximate mesh-based Ewald methods^12–16^) has long appeared as a necessity to increase computational efficiency. It is, however, artificial. On the one hand, its long-range nature translates the physics of the London dispersion force^17^. On the other hand, the phase diagram of the model depends strongly on the cutoff^18,19^, so that results at different cutoffs have, in principle, to be extrapolated carefully. In the Lennard-Jones liquid, the surface tension and the Tolman length depend on the cutoff^20,21^. The long-range nature of the Coulomb potential, which must be preserved^11^, is usually treated through fast mesh-based Ewald methods^12–16^ that solve the Poisson equation in discretized reciprocal space. The error of the computation and, thus, of the sampling of configurations, depends on a target-precision parameter that, in principle, calls for a costly extrapolation in order to be eliminated. Beyond their inherent approximations, cutoffs, discretizations of reciprocal space, and thermostats can even introduce unphysical artifacts in molecular-dynamics simulations^11,12,22^ that complex algorithms aim to keep under control.

Our alternative paradigm for the molecular simulation of static properties in thermodynamic equilibrium is based on several modern concepts in statistics^23,24^, namely the factorized Metropolis filter^25^, the concept of thinning^26^ as expressed in the cell-veto algorithm^27^, that of continuous-time piecewise-deterministic Markov processes^28^ as realized in the event-chain Monte Carlo algorithm^25,29,30^ and, finally, Walker’s method of aliases^31^ that can be adapted to the fast sampling of long-range interactions^27,32^. The paradigm is rigorously exact from the start and, by construction, strictly samples the canonical ensemble without thermostats. Deterministic trajectories of atoms in continuous Monte-Carlo time follow an ordinary differential equation that can be integrated analytically. Trajectories are interrupted by *events* at random times. This non-reversible piecewise-deterministic Markov process violates the detailed-balance condition normally associated with thermal equilibrium, but still samples the Boltzmann distribution at each moment of its continuous-time evolution.

A particular algorithm that generates non-reversible Markov processes, event-chain Monte Carlo, has led to spectacular speedups of local Markov chains in statistical physics^33^. In molecular systems, short- and long-range potentials are handled without any cutoffs or discretizations and, as we show in this paper, with competitive efficiency. The Boltzmann weight $\pi =\exp (-\beta U)$ (with $\beta$ the inverse temperature and *U* the potential) is expressed as a factorized product $\pi =\prod _M \exp (-\beta U_M)$ of statistically independent factors *M* with factor potentials $U_M$ with $\sum _M U_M = U$ that each depend only on a small subensemble of atoms^25^. Every factor stochastically generates a candidate event time when the piecewise-deterministic motion must be interrupted. The minimum of these times triggers an event, and determines the initial conditions for the next piece. The total potential *U* and the corresponding forces never need evaluating, yet the stationary state is rigorously the Boltzmann distribution. This circumvents the problem of the inaccurate evaluation of the long-range forces between all atoms in the molecular-dynamics approach that is nowadays mostly used even for static properties in thermodynamic equilibrium. In the context of molecular simulation, the paradigm of non-reversible and event-driven Markov processes was up to now a mere theoretical possibility that converge only for the tiniest molecular systems^23,24^.

The present paper is based on essential new developments in the event-driven paradigm. In particular, we generalize a particular non-reversible Markov-chain Monte Carlo algorithm for hard spheres, Newtonian event-chain Monte Carlo^30^, to the factor potentials of molecular simulation. We implement this modified algorithm in demonstration software for *N* flexible SPC/Fw water molecules^34^ interacting with the long-range Coulomb potential. As a benchmark, we concentrate on sampling the electric polarization^35^ (the electric dipole moment of the simulation cell). Fluctuations in this quantity determine the dielectric properties of water. For large system sizes, we show that our demonstration software confirms the theoretical expectation^23^ that the non-reversible Markov process decorrelates the polarization in a computer time that scales as $N \log N$, similar to state-of-the-art mesh-based Ewald methods in the molecular-dynamics approach^13^ but without a prefactor that diverges with the target precision for the force evaluations. Moreover, the generalized Newtonian event-chain Monte Carlo algorithm greatly reduces autocorrelation times compared to the previously considered event-chain Monte Carlo variant^23^. This allows us to actually equilibrate large molecular systems. We show that our non-reversible algorithm overcomes the slow diffusive dynamics of reversible Monte Carlo algorithms, while the factorization does not penalize the dynamics in comparison to the molecular-dynamics approach. In terms of computer time, our approximation-free code reaches a break-even point with respect to a standard molecular-dynamics code that is run at a different target precisions for the long-range Coulomb interaction below machine precision. The considerable performance difference between event-chain Monte Carlo variants evidences the greater algorithmic freedom in our non-reversible Markov-process approach compared to the molecular-dynamics approach. Furthermore, we point out how our demonstration software can be improved in the future. Practical molecular simulations based on our paradigm are within reach, and we set out an interdisciplinary research strategy for sampling the Boltzmann distribution without any bias and for using non-reversible Markov chains as a gold standard for molecular simulation, in particular in the presence of long-range interactions.

## Results

### Modern-statistics paradigm for SPC/Fw water

In a molecular system with long-range interactions, the force on an atom depends on the position of all other atoms, rendering its evaluation tedious unless one introduces cutoffs or one discretizes reciprocal space. In contrast, we implement a piecewise-deterministic Markov process through the event-chain Monte Carlo algorithm, where a single atom moves with constant velocity at any given moment^25,29,36^. The deterministic motion of this atom is interrupted by an event that stops it and sets off a similar motion of a new atom. Factors *M* in the SPC/Fw water model describe O–H bonds, the bending of H–O–H opening angles, O–O intermolecular Lennard-Jones interactions, and the Coulomb interaction between two water molecules (see Methods section). In a non-homogeneous Poisson process, each factor *M* proposes an independent *candidate event time* where an exponentially distributed random number is equal to the integrated *factor event rate*, that is, the cumulative increments of the factor potential $U_M$ induced by the moving atom [see Eqs. (1) and (2) in the Methods section]. The minimum over all the candidate event times then realizes the next event, and motion is transferred to another atom contributing to the corresponding factor potential $U_M$. This succession of events takes place in continuous Monte Carlo time, and the Boltzmann distribution is sampled at all times^28,36^. In comparison to the usual Metropolis algorithm^37^, our formulation replaces rejections by transfers of motion.

**Figure 1:**
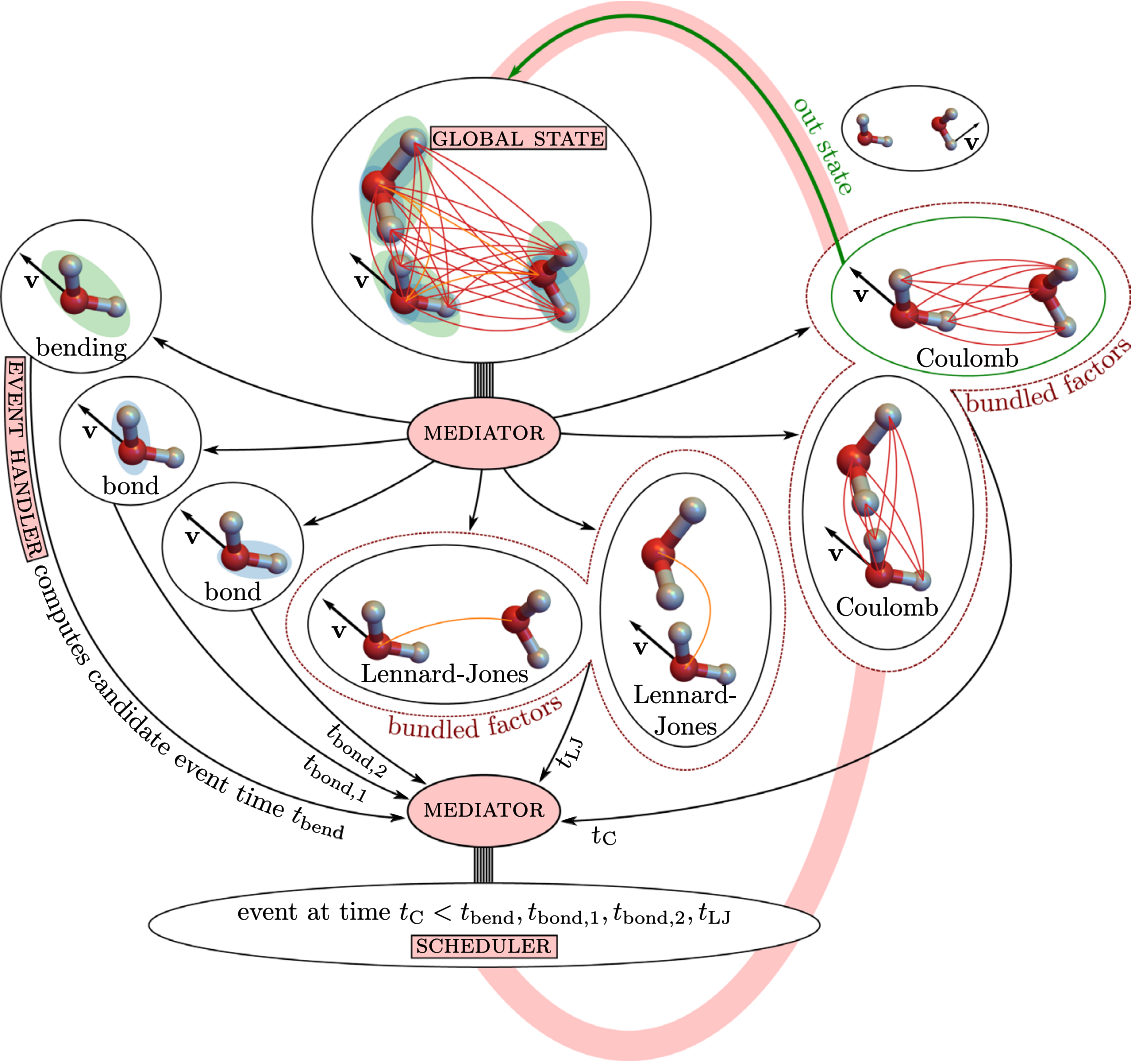
jellyfysh implementation of our Markov-chain paradigm. The *mediator* splits the *global state* into statistically independent factors. Factors communicate independent candidate event times, the earliest of which defines the next event. Factors for long-range interactions are bundled using the cell-veto algorithm, so that the number of *event handlers* remains limited. Candidate event times are collected by the mediator and then treated in the *scheduler*. The factor triggering the event then updates the global state, again *via* the mediator.

In our implementation of the non-reversible Markov-chain paradigm in the jellyfysh application^24^, positions and velocities of atoms define the *global state* of the physical system. To impose coherency of the physical system, the global state is accessed only through a central *mediator*^38^ that dispatches physically independent computations of candidate events to *event handlers*. A *scheduler* weeds through candidate events. It identifies the unique event and the corresponding event handler that provides the subsequent transfer of motion, leading to an update of the global state (see Fig. 1). The event handlers within the mediator architecture mirror the statistical independence of the factors composing the physical system. This allows us to compose complex interactions in a transparent and independent manner. The pseudocode in Algorithm 1 in the Methods section summarizes the general implementation of an event computation within jellyfysh (including the cell-veto algorithm that is described in the next section).

A number of inequivalent options have been constructed for the update of active particles within event-chain Monte Carlo^23,39^. Similar flexibility is possible in the updating of velocities^29,30,40,41^, as well as in parallelizing the algorithm^24,42^. The original straight variant is most efficient for simple hard disks^29,33^, but is completely inappropriate for hard-disk dipoles^41^ and water molecules^43^ that require the exploration of internal rotational degrees of freedom. In this paper, we generalize Newtonian event-chain Monte Carlo^30^ from systems akin to the hard-disk system to molecular systems (see Methods section). It requires no fine-tuning and again exactly samples the canonical Boltzmann distribution. For the SPC/Fw water model, this paper shows that generalized Newtonian event-chain Monte Carlo is much more efficient than the fine-tuned straight variant. Piecewise-deterministic Markov processes offer an even wider choice of options for the management of events, the selection of factors, and the piecewise-deterministic trajectories, that may well apply to molecular simulation in the approximation-free non-reversible Markov-chain framework and may achieve further unforeseeable speedups.

### Implementation for SPC/Fw water: Cell-veto—Fibonacci sphere

**Figure 2:**
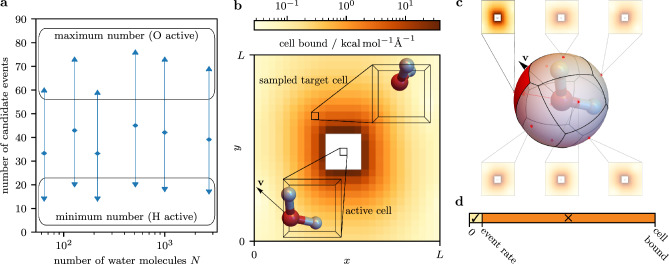
Long-range interactions with constant computer time per event. (**a**) The number of candidate events (event handlers) is constant for increasing system sizes. (**b**) Walker table from which a target cell is sampled according to its cell bound with respect to the active cell containing the moving atom. (**c**) Different Walker tables for Fibonacci vectors on the unit sphere. The active atom obtains cell bounds from the nearest vector. (**d**) The thinning procedure confirms (✓) or rejects (✕) the event using the actual factor event rate of the molecules in the active and target cell.

In the event-driven implementation of our method (see Fig. 1), a single atom moves among the other atoms and molecules, so that $\mathcal {O}(N)$ long-range factors are changing with time and, in principle, yield independent events that would require sorting and managing. The cell-veto algorithm, as discussed below, allows one to bundle most of the long-range Coulomb and Lennard-Jones factors and, in the SPC/Fw water model, the mediator interacts with only $\sim 50$ event handlers that propose candidate event times to the scheduler (see Fig. 2a). This number contains the two event handlers for the bundled long-range factors. In addition, it contains the event handlers for the bond and bending factors, and for the Coulomb and Lennard-Jones factors between nearby water molecules that were specifically excluded to minimize computer time (see Methods section). The bundling allows the processing of each event in constant computer time (for large *N*) while treating the long-range interactions without approximations.

In our context of the SPC/Fw water model, the cell-veto algorithm^27^ upper-bounds the factor event rates for pairs of molecules interacting with the Coulomb potential by precomputed, time-independent bounds for these molecules somewhere within a pair of cells (see Fig. 2b). The full set of these *cell bounds* corresponds to the set of bundled factors of the long-range interaction. Walker’s method of aliases^31^ conserves the cell bounds in a *Walker table*. In the event-driven evolution of the piecewise-deterministic Markov process, the set of cell bounds in the Walker table provides a single candidate event time for the entire set of bundled factors, and Walker’s method samples an associated single cell bound—and thus a single associated factor—with constant algorithmic complexity. The overestimation of the factor event rate by the cell bound is exactly corrected in a procedure akin to the thinning of non-homogeneous Poisson processes^26^ by confirming the transfer of motion in the event (see Fig. 2d and Algorithm 1). This thinning is performed with the actual positions of the atoms and the actual velocity of the moving atom, leading to an exact treatment of the long-range interaction that is independent of the set of cell bounds (see Ref.^44^ for a simple example on how the thinning procedure exactly corrects for the overestimation of the cell bounds with respect to the underlying factor event rates).

The factor event rates, and hence the cell bounds, depend on the velocity of the moving atom. In all previous applications of the cell-veto algorithm in conjunction with the original straight event-chain Monte Carlo variant, there was only a small number of possible velocities and a Walker table was built for each of them^23,24,27^. In order to use the cell-veto algorithm with our generalized Newtonian event-chain Monte Carlo variant, we discretize its continuous velocity space for the precomputation of the cell bounds. We build separate Walker tables for multiple directions of the velocity of the moving atom corresponding to *Fibonacci vectors* on the unit sphere (see Fig. 2c and Methods section). During the simulation, the actual velocity of the moving atom is mapped to the closest Fibonacci vector. The thinning procedure again exactly corrects for this discretization of velocity space.

### Benchmarking for SPC/Fw water

**Figure 3:**
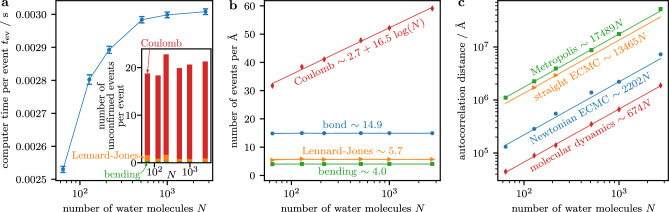
Event rates and decorrelation in the SPC/Fw water model. (**a**) Computer time per event in jellyfysh. Inset: Number of unconfirmed events per event for different factor types. (**b**) Event rate in jellyfysh for different factor types. (**c**) Distance to decorrelate the polarization for different sampling algorithms (for molecular dynamics: sum over the average displacements of all atoms per time step).

For our benchmark, we release a new major version of the jellyfysh application^24^ that implements generalized Newtonian event-chain Monte Carlo and, consequently, the cell-veto algorithm with a discretized velocity space. Only the new version actually succeeds in equilibrating large configurations of *N* SPC/Fw water molecules in a periodic box at standard density and temperature. We implement long-range molecular Coulomb factors with Walker tables that we also adopt for the Lennard-Jones interaction (see Methods section for details). We find that generalized Newtonian event-chain Monte Carlo, for large *N*, requires a computer time per event that remains constant (see Fig. 3a). A large number of unconfirmed events stems from the overestimated cell bounds which, e.g., do not account for the relative orientation of molecules (see inset of Fig. 3a). Since every unconfirmed event requires a high-precision evaluation of a long-range interaction between two water molecules, reducing this number will much reduce computer times. While the computer time per event is constant, the number of events per Ångström (that is, per unit Monte-Carlo time) increases logarithmically with *N* for the Coulomb factors (see Fig. 3b), as predicted by theory^23^. In summary, our approach requires a computer time scaling as $N \log N$ to advance *N* water molecules by a constant distance. This matches the complexity of mesh-based Ewald methods in the molecular-dynamics approach^13^, but without their slowdown as the target precision is increased because jellyfysh treated long-range interactions exactly to begin with.

For concreteness, we compare the decorrelation of the polarization of the water system within jellyfysh to molecular-dynamics simulations within the lammps software^8^ on a single processor with default parameters and a ${1}\,{\hbox {fs}}$ time step (see Methods section). To decorrelate this local quantity, both lammps and jellyfysh must move the atoms of any water molecule by a characteristic distance (see Fig. 3c and Methods section for details). This characteristic distance lacks a clear physical interpretation for the Metropolis and event-chain Monte Carlo algorithms because of their *ad-hoc* dynamics for exploring sample space. However, it provides a fair measure of the distance in sample space that separates independent samples (“lower” means “faster”). Different variants of event-chain Monte Carlo vary in their efficiency, and our generalized Newtonian variant is an order of magnitude faster than the original straight variant as the characteristic distance is reduced from 13465 Å to 2202 Å. In comparison to molecular dynamics with the smallest characteristic distance of 674 Å, the non-reversible Markov process only moves a single atom at any point in time which may explain the slightly larger characteristic distance of generalized Newtonian event-chain Monte Carlo. The reversible Metropolis algorithm with analogous single-atom moves (that, in principle, also reaches an $N\log N$ scaling^45^ by using a recent variant of the fast multipole method^46^), as implemented in the dl_monte software package^47^, is clearly inferior to our non-reversible methods with a characteristic distance of 17489 Å.

**Figure 4:**
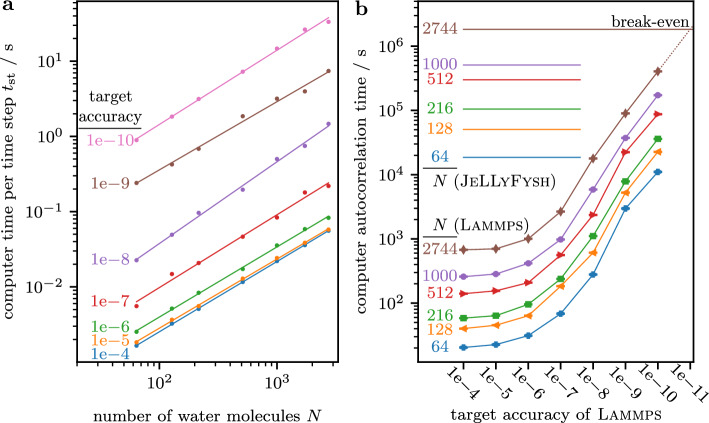
lammps–jellyfysh benchmark for the SPC/Fw water model. (**a**) Computer time per step of lammps for different target accuracies of its particle–particle particle–mesh solver. (**b**) Computer time used by lammps to decorrelate the polarization depends on the target accuracy and the number of water molecules *N*. jellyfysh is exact up to machine precision. The break-even precision is indicated.

In the molecular-dynamics simulations that we benchmark our approach against, we use a state-of-the-art mesh-based Ewald method to treat the long-range Coulomb interaction. Its implementation in lammps sets a target accuracy based on analytic error estimates obtained from a specific charge distribution^15,48,49^. To accelerate the evaluation of the reciprocal sum in the Ewald method, the charges of atoms (which live in continuous space) are mapped onto a grid using an interpolation scheme. Finer grids and higher interpolation orders yield higher target accuracies. For various system sizes, we estimate the required computer times for different target accuracies by changing the grid spacing, using the lammps defaults. Naturally, higher target accuracies require more computer time (see Fig. 4a). Because of the underlying factorization of the Boltzmann distribution, event-chain Monte Carlo is rigorously approximation-free. It only explicitly considers the Coulomb interaction between two SPC/Fw water molecules (mostly when candidate events from the cell-veto algorithm are confirmed). Our implementation in jellyfysh uses the historic Ewald summation in continuous space for this interaction between a small number of charges that stays constant with increasing system size. We tune it to machine precision without any assumptions on the global charge distribution. In comparison to the molecular-dynamics approach at different target accuracies for the long-range Coulomb interaction as implemented in lammps, our non-reversible piecewise-deterministic Markov process, specifically the generalized Newtonian event-chain Monte Carlo as implemented in jellyfysh, reaches the break-even point well below machine precision (see Fig. 4b).

## Discussion

In this paper, we benchmarked an implementation of a modern-statistics paradigm for molecular simulations of static properties in thermodynamic equilibrium in the standard SPC/Fw water model. The time dependence of the corresponding Markov process differs from the physical dynamics yet it exactly approaches thermal equilibrium on time scales that are potentially faster than in nature. Its remarkable efficiency (that we expressed as an $N \log N$ computer time to decorrelate a local observable, the polarization) is rooted in three paradoxes. First, the Markov process is non-reversible (that is, effectively out-of-equilibrium), yet its steady state coincides with the equilibrium Boltzmann distribution^50^. In contrast to standard Monte Carlo algorithms that satisfy the detailed-balance condition and only move diffusively, it features finite probability flows, making it capable of moving ballistically. This has already led to considerable speedups in a variety of fields ranging from physics to statistics and machine learning (see, e.g., Refs.^36,51–53^). In statistical mechanics, a decrease in the dynamical scaling exponents was demonstrated in a number of interacting particle models in one and higher dimensions, resulting in speedups with respect to reversible Markov chains and to molecular dynamics that diverge with the system size^54,55^. It remains to be seen whether non-local observables as, for example, large-scale hydrodynamic modes, macroscopic conformations, and order parameters can similarly, in chemical physics, benefit from non-reversibility. The second paradox in our approach is that the Boltzmann distribution $\exp (-\beta U)$ is sampled without any approximation and with great efficiency although the total potential *U* and its derivatives, the forces, are never evaluated. This sidesteps all the problems with limited-precision calculations of energies and forces. The third paradox is the bundling of $\mathcal {O}(N)$ independent decisions to interrupt the straight-line trajectory of the piecewise-deterministic Markov process into an expression that can be evaluated in constant time. The Walker tables bundle a multitude of long-range Coulomb factors and of Lennard-Jones factors into two candidate events, one for long-range Coulomb, one for long-range Lennard-Jones, which allows us to handle a complex decision (a conjunction of $\mathcal {O}(N)$ factor-wise decisions of independent factor potentials $U_M$) in a few operations, even in the $N \rightarrow \infty$ limit.

Our software jellyfysh is openly available and fully functional, although it has only the status of demonstration software. It implements the interactions of SPC/Fw water, and provides all necessary configuration files to replicate the simulations of this paper out-of-the-box. In the Methods section, we further provide the detailed simulation protocol of event-chain Monte Carlo in jellyfysh. The flexible mediator-based architecture of jellyfysh allows for simple extensions to molecular systems beyond water molecules. Although it is still mostly written in the relatively slow Python language, the new major version that we have described in this paper is of practical use for molecular simulation. Our method is exact from the very beginning and our application becomes competitive with the traditional molecular-dynamics code lammps at high intrinsic precision. Optimizing jellyfysh in the same way that lammps was optimized over decades will greatly reduce the break-even point. In addition, future research will be able to concentrate on the most efficient ones among a large choice of cell bounds, factorizations, Fibonacci vectors and variants of the piecewise-deterministic Markov processes. The most efficient application to non-homogeneous systems and to non-homogeneous (for example, corrugated) boundary conditions will be other interesting directions of research. The parallelization of our method to a large number of active particles has for the moment been implemented only for hard-disk potentials^42^. The generalization to arbitrary potentials presents an outstanding challenge. Clearly, more interdisciplinary research from statistics to computational chemistry will clarify whether all of this provides a sufficiently strong basis for an alternative approach for practical molecular simulation.

With its guarantee for the unbiased sampling of the Boltzmann distributions, our paradigm may serve as a gold standard for molecular simulation in general, capable of identifying artifacts and approximations that may not have been totally eliminated through the symptomatic algorithmic approach in molecular-dynamics simulations. Furthermore, given the greater algorithmic freedom for the Markov-chain approach than for the molecular-dynamics approach, it may actually become faster than molecular dynamics to explore thermodynamic equilibrium. Several orders of magnitude in numerical speed can certainly be gained by re-engineering our software, which would then be able to tackle the peptide-in-water benchmark problem^2^ that has had a major influence over the last decade. The great simplicity of our approach and its present implementation in the jellyfysh software may well facilitate further developments. Ultimately, our paradigm may yield independent equilibrium samples that then serve, in synergy with long-established molecular-dynamics software, as starting configurations for parallel molecular dynamics calculations in order to access high-precision dynamical correlation functions.

## Methods

### Generalized Newtonian event-chain Monte Carlo

Event-chain Monte Carlo is a family of local non-reversible Markov-chain Monte Carlo algorithms that implement piecewise-deterministic Markov processes. All variants are event-driven and move a single atom along a straight-line trajectory at any time. They differ only in their update of the moving atom and its velocity in the transfers of motion at event times. Straight event-chain Monte Carlo is the original variant and proved to be most efficient for the hard-disk model^29,33^. Its generalization to the translationally invariant factor potentials of molecular systems^23,25^ is implemented in the version 1.0 of jellyfysh^24^.

We develop version 2.0 of jellyfysh^56^ for this paper. The pseudocode in Algorithm 1 provides a high-level summary of its general implementation of a piecewise-deterministic Markov process. This pseudocode complements the mathematical description in the following, and Fig. 5 in Ref.^24^ that introduced the basic architecture of jellyfysh. Besides the straight variant, version 2.0 of jellyfysh also implements the generalized Newtonian event-chain Monte Carlo algorithm. Newtonian event-chain Monte Carlo was originally only formulated for the hard-sphere model that contains peculiar stepwise-changing two-body factor potentials^30^. Among various event-chain Monte Carlo variants, it was shown to escape faster from sparse hard-disk packings^57^ and to produce favorable rotation dynamics in tethered hard-disk dipoles^41^. Its superiority is confirmed for the rotation dynamics of SPC/Fw water molecules in this paper, but requires its generalization to smooth factor potentials that depend on an arbitrary number of atom positions.

**Algorithm 1:**
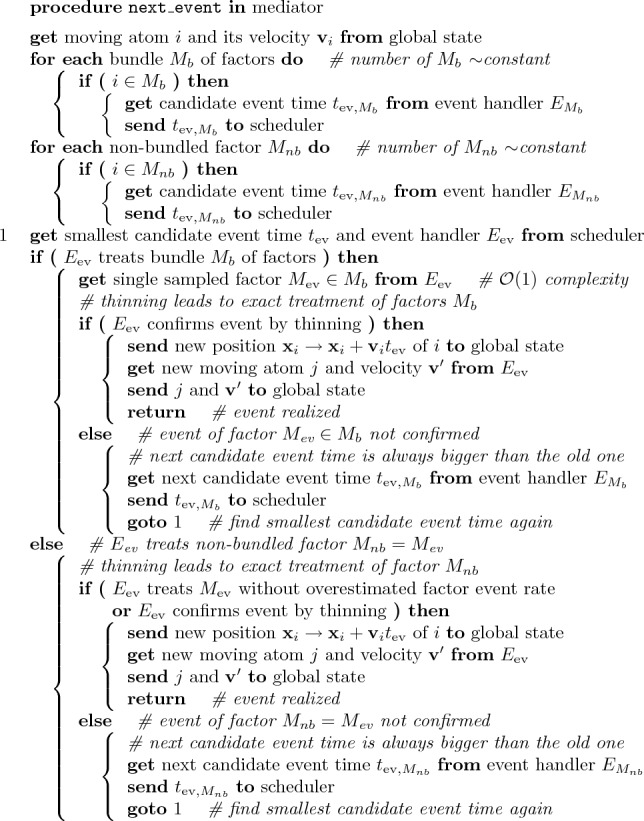
Procedure that realizes the next event in the jellyfysh implementation. The *mediator* communicates with the *global state*, the *event handlers*, and the *scheduler*. Factors for long-range interactions are bundled to achieve a constant number of event handlers that compute candidate event times. Thinning exactly corrects for candidate event times that were computed based on overestimated factor event rates.

We follow the initial introduction of non-reversible event-chain Monte Carlo as a lifted continuous-time Markov process^25^ (see also the formulation as a piecewise-deterministic Markov process in Ref.^58^). Consider $N_a$ atoms and let $\textbf{x}= (\textbf{x}_1, \ldots , \textbf{x}_{N_a}) \in \mathbb {R}^{3N_a}$ be the physical all-atom configuration that collects all three-dimensional atom positions $\textbf{x}_i\in \mathbb {R}^3$ with $i\in \{1,\ldots ,N_a\}$. For generalized Newtonian event-chain Monte Carlo, the lifting framework^59,60^ (see also Refs^36^ and^61^, Appendix A) extends the physical all-atom configuration $\textbf{x}$ to $(\textbf{x}, \textbf{v}, i)$, with auxiliary lifting variables that represent an all-atom velocity $\textbf{v}$ and an activity label *i*, respectively. The physical sample space $\textbf{x}\in \Omega$ is extended to the lifted sample space $(\textbf{x}, \textbf{v}, i) \in \widehat{\Omega } = \Omega \times \mathcal {V}^{3N_a}(E_\text {kin}) \times \mathcal {N}$, where $\mathcal {N}=\{1,\ldots , N_a\}$ is the set of atom indices, and $\mathcal {V}^{3N_a} (E_\text {kin}) = \{ \textbf{v}\in \mathbb {R}^{3N_a}: \textbf{v}^T \, \textbf{M} \, \textbf{v}= 2E_\text {kin} \}$ with a positive-definite symmetric mass matrix $\textbf{M}$ and conserved total kinetic energy $E_\text {kin}$. The Markov process targets the lifted stationary distribution $\hat{\pi }(\textbf{x},\textbf{v},i) = \pi (\textbf{x}) \times \mu _\mathcal {V}(\textbf{v}) \times \mu _\mathcal {N}(i)$ that separates into the factorized Boltzmann distribution $\pi (\textbf{x}) \propto \exp [-\beta U(\textbf{x})]=\prod _M \exp [-\beta U_M(\textbf{x})]$, and the uniform distributions $\mu _\mathcal {V}(\textbf{v})$ on $\mathcal {V}^{3N_a}$, and $\mu _\mathcal {N}(i)$ on $\mathcal {N}$. Since the kinetic energy is fixed throughout a simulation of generalized event-chain Monte Carlo, its kinetic-energy distribution does not coincide with that of the canonical ensemble. The all-atom “velocity” lacks a physical meaning.

Given a lifted configuration $(\textbf{x}(t_0), \textbf{v}(t_0), i)$ at time $t_0$, Newtonian event-chain Monte Carlo continuously moves the single active atom *i* starting from its position $\textbf{x}_i(t_0)$ with its constant velocity $\textbf{v}_i = \textbf{v}_i(t_0)$ up to an event at time $t_\text {ev}>t_0$, which interrupts the motion: $\textbf{x}_i(t) = \textbf{x}_i(t_0) + \textbf{v}_i \, (t - t_0)$ for $t_0\leq t < t_\text {ev}$. The velocities $\textbf{v}_j$ of all other atoms $j\ne i$ in the all-atom velocity $\textbf{v}$ are, at this point, hypothetical, that is, mere labels. The time-dependent event rate $\lambda (t)$, that is, the probability density to interrupt the piecewise-deterministic motion of the active atom *i*, is given by a sum of factor event rates $\lambda _M(t)$:

1 $$\begin{aligned} \lambda (t) = \sum _M \lambda _M(t) = \sum _{M} \beta \max \left[ 0,\varvec{\nabla }_{\textbf{x}_i} U_M\left( \textbf{x}(t)\right) \cdot \textbf{v}_i\right] . \end{aligned}$$

All factors *M* can be considered as statistically independent. Each of them stochastically generates a candidate event time $t_{\text {ev},M}$ in an inhomogeneous Poisson process based on its factor event rate $\lambda _M(t)\ge 0$ (that is only nonzero for factor potentials $U_M$ that are actually changed by the motion of the active atom):

2 $$\begin{aligned} {{\,\textrm{ran}\,}}_M(0,1) = \exp \left[ - \int _{t_0}^{t_{\text {ev}, M}}\lambda _M(t)\, \textrm{d}t\right] . \end{aligned}$$

Here, ${{\,\textrm{ran}\,}}_M(0,1)$ is a uniformly distributed random number between 0 and 1 that is drawn separately for each factor *M*. In Eq. (2), the cumulative increments of the factor potential $U_M$ under the motion of the active atom with its velocity $\textbf{v}_i$ since $t_0$ are equal to an exponentially distributed random number with mean $1/\beta$ at the time $t_{\text {ev},M}$. If the computation of $t_{\text {ev}, M}$ from Eq. (2) is tedious or even impossible, a Poisson thinning procedure replaces the factor event rate $\lambda _M(t)$ by an upper bound $\tilde{\lambda }_M(t)$ that satisfies $\tilde{\lambda }_M(t)\ge \lambda _M(t)$ for all *t*^26,36^. Superfluous events from the increased factor event rate are thinned out by first confirming an event at the sampled candidate event time $t_{\text {ev},M}$ with probability $\lambda _M(t_{\text {ev},M}) / \tilde{\lambda }_M(t_{\text {ev},M})\le 1$.

At the (confirmed) event time $t_\text {ev}=\min _M t_{\text {ev}, M}$, the motion of the active atom *i* is interrupted at the lifted configuration $(\textbf{x}= \textbf{x}(t_\text {ev}), \textbf{v},i)$. An event changes the lifting variables and sets the initial lifted configuration $(\textbf{x}, \textbf{v}', j)$ for the next piece in the piecewise-deterministic Markov process. The event is realized by a unique event factor $M_\text {ev} = \mathop {\mathrm {arg\,min}}_Mt_{\text {ev}, M}$. Generalized Newtonian event-chain Monte Carlo proposes an update of the all-atom velocity $\textbf{v}\rightarrow \textbf{v}'$ at time $t_\text {ev}$ with a “force kick” in the direction of the gradient of the event-factor potential:

3 $$\begin{aligned} \textbf{v}' = \textbf{v}- 2 \, \frac{\textbf{v}\cdot \varvec{\nabla }_{\textbf{x}} U_{M_\text {ev}}}{(\varvec{\nabla }_{\textbf{x}}U_{M_\text {ev}})^T\, \textbf{M}^{-1} \,\varvec{\nabla }_{\textbf{x}}U_{M_\text {ev}}} \, \textbf{M}^{-1}\,\varvec{\nabla }_{\textbf{x}} U_{M_\text {ev}}. \end{aligned}$$

This force kick leaves the kinetic energy $2E_\text {kin} = \textbf{v}^T \, \textbf{M} \, \textbf{v}$ invariant and applying it twice yields the original $\textbf{v}$^62^. The event-factor potential only acts on a small subensemble $k\in M_\text {ev}$ of atoms, and thus only the velocities $\textbf{v}_{k}$ of these contributing atoms are possibly modified. For the simple choice of an identity mass matrix $\textbf{M} = \textbf{I}$, Eq. (3) may be written as

4 $$\begin{aligned} \textbf{v}_k' = \textbf{v}_k - 2 \, \frac{\sum _{j\in M_\text {ev}} \textbf{v}_j \cdot \varvec{\nabla }_{\textbf{x}_j} U_{M_\text {ev}}}{\sum _{j\in M_\text {ev}} \left| \varvec{\nabla }_{\textbf{x}_j} U_{M_\text {ev}} \right| ^2}\, \varvec{\nabla }_{\textbf{x}_k} U_{M_\text {ev}}. \end{aligned}$$

In generalized Newtonian event-chain Monte Carlo, only a single active atom moves with its velocity at any time. At an event, it must choose the next active atom $k\in M_\text {ev}$ from the event factor. It also chooses to either change the velocity $\textbf{v}\rightarrow \textbf{v}'$ of all contributing atoms according to Eq. (3), or to keep the all-atom velocity constant $\textbf{v}\rightarrow \textbf{v}$. Generalized Newtonian event-chain Monte Carlo offers two schemes to treat events. The *Newtonian-general* scheme applies to general mass matrices $\textbf{M}$ and factors that depend on an arbitrary number of atoms. The *Newtonian-pair* scheme only applies to distance-dependent pair potentials and an identity mass matrix $\textbf{M}=\textbf{I}$.

In the Newtonian-general scheme, the probability for each possible choice $(k,\textbf{v}')$ or $(k, \textbf{v})$ of the lifting variables is given by $\max [0, -\varvec{\nabla }_{\textbf{x}_k} U_{M_\text {ev}} \cdot \textbf{v}_k'] / C$ or $\max [0, -\varvec{\nabla }_{\textbf{x}_k} U_{M_\text {ev}} \cdot \textbf{v}_k] / C$, respectively. Here, *C* is a common normalization factor. If the force kick is not applied and $\textbf{v}$ stays constant, the active atom always changes because the factor event rate was positive at the time of the event [see Eqs. (1) and (2)]. Likewise, if the event-factor potential only depends on a single atom (as for external potentials), the force kick is always applied.

In the Newtonian-pair scheme for distance-dependent pair potentials between two atoms *a* and *b*, $U_{M_\text {ev}} = U_{M_\text {ev}}(|\textbf{x}_a - \textbf{x}_b|)$, and an identity mass matrix $\textbf{M}=\textbf{I}$, we alternatively exploit the inherent translational invariance to treat the event deterministically. We always apply the force kick in Eq. (4) to modify both $\textbf{v}_a$ and $\textbf{v}_b$, and always change the active atom within the event factor. In this case, Eq. (4) is equivalent to an elastic Newtonian collision between two particles of equal mass. This alternative scheme recovers the original formulation of Newtonian event-chain Monte Carlo for the hard-sphere model^30^.

Samples of all-atom configurations $\textbf{x}$ that can be used to compute observables are taken at periodic time intervals^30^. Furthermore, Newtonian event-chain Monte Carlo requires resampling of the all-atom velocity $\textbf{v}$ and the active atom *i* at periodic time intervals $\tau _\text {chain}$ in order to be irreducible^41^. We sample $\textbf{v}$ and *i* from their respective stationary distributions $\mu _\mathcal {V}(\textbf{v})$ and $\mu _\mathcal {N}(i)$. For the all-atom velocity $\textbf{v}$, this is equivalent to sampling a multivariate Gaussian distribution with mean $\varvec{\mu } = \textbf{0}$ and covariance matrix $\varvec{\Sigma } = \textbf{M}^{-1}$, followed by corrections of the sampled total velocity so that $\textbf{1} \cdot \textbf{v}{=} 0$ and $\textbf{v}^T \, \textbf{M} \, \textbf{v}{=} 2 E_\text {kin}$. The activity label *i* is sampled uniformly from the set $\mathcal {N} = \{1 \ldots N_a\}$.

### Proof of correctness of generalized Newtonian event-chain Monte Carlo

In order to prove the correctness of the Newtonian event-chain Monte Carlo algorithm for general smooth interactions, we show that the global balance condition is satisfied. The total probability flow into any lifted configuration $(\textbf{x}, \textbf{v}, i)\in \widehat{\Omega }$ consists of a physical flow $\mathcal {F}^\text {phys}(\textbf{x}, \textbf{v}, i)$ and a lifting flow $\mathcal {F}^\text {lift}(\textbf{x}, \textbf{v}, i)$, and must equal its statistical weight $\hat{\pi }(\textbf{x}, \textbf{v}, i)$:

5 $$\begin{aligned} \hat{\pi }(\textbf{x}, \textbf{v}, i) = \mathcal {F}^\text {phys}(\textbf{x}, \textbf{v}, i) + \mathcal {F}^\text {lift}(\textbf{x}, \textbf{v}, i). \end{aligned}$$

The physical flow into $(\textbf{x}, \textbf{v}, i)$ stems from the continuous movement of the active atom *i* that was not interrupted by an event with the event rate given in Eq. (1). With $\textbf{x}' = (\textbf{x}_1, \ldots , \textbf{x}_i - \textbf{v}_i \textrm{d}t, \ldots , \textbf{x}_{N_a})$, we get

6 $$\begin{aligned} \begin{aligned} \mathcal {F}^\text {phys}(\textbf{x}, \textbf{v}, i)&= \hat{\pi }(\textbf{x}', \textbf{v}, i) \left\{ 1 - \beta \sum _M \max \left[ 0, \varvec{\nabla }_{\textbf{x}'_i} U_M \cdot \textbf{v}_i \right] \right\} \\&= \hat{\pi }(\textbf{x}, \textbf{v}, i) \left\{ 1 - \beta \sum _M \max \left[ 0, -\varvec{\nabla }_{\textbf{x}_i} U_M \cdot \textbf{v}_i\right] \right\} \\&= \hat{\pi }(\textbf{x}, \textbf{v}, i) + \sum _M \mathcal {F}^\text {phys}_M (\textbf{x}, \textbf{v}, i), \end{aligned} \end{aligned}$$

where the second line uses the detailed-balance property of the factorized Metropolis filter that yields the event rates in Eq. (1) and allows treating all factors as being statistically independent^25^. The lifting flow into $(\textbf{x}, \textbf{v}, i)$ stems from interrupted motions of lifted configurations $(\textbf{x}, \textbf{v}', j)$ with different lifting variables $\textbf{v}'$ and *j* but the same physical all-atom configuration $\textbf{x}$:

7 $$\begin{aligned} \begin{aligned} \mathcal {F}^\text {lift}(\textbf{x}, \textbf{v}, i)&= \beta \sum _M\sum _{j\in \mathcal {N}} \int _{\mathcal {V}^{3N_a}(E_\text {kin})} \textrm{d}^{3N_a}\textbf{v}'\, \hat{\pi }(\textbf{x}, \textbf{v}', j) \max \left[ 0, \varvec{\nabla }_{\textbf{x}_j}U_M\cdot \textbf{v}'_j\right] p^M_{(\textbf{v}',j),(\textbf{v}, i)} \\&= \sum _{M} \mathcal {F}^\text {lift}_M(\textbf{x}, \textbf{v}, i). \end{aligned} \end{aligned}$$

Here, $p_{(\textbf{v}',j), (\textbf{v}, i)}$ is the probability to change the lifting variables $(\textbf{v}', j)$ to $(\textbf{v},i)$. For every factor *M*, the lifting flow $\mathcal {F}^\text {lift}_M(\textbf{x}, \textbf{v}, i)$ in Eq. (7) exactly cancels the physical flow $\mathcal {F}^\text {phys}_M(\textbf{x}, \textbf{v}, i)$ in Eq. (6) so that the global-balance condition in Eq. (5) is satisfied. A trivial (inefficient) solution would be to simply invert the velocity of the active atom *i* in an event while keeping it active: $p^M_{(\textbf{v}',j),(\textbf{v},i)} = \delta _{ij} \, \delta ^{(3)}(\textbf{v}_i - \textbf{v}'_i)$.

We presented two schemes for the update of the lifting variables in an event of generalized Newtonian event-chain Monte Carlo. The first stochastic Newtonian-general scheme applies for a general number of atoms on the event factor. The second deterministic Newtonian-pair scheme exploits the translational invariance of distance-dependent pair potentials. For the Newtonian-general scheme, we get

8 $$\begin{aligned} \begin{aligned} p^M_{(\textbf{v}', j), (\textbf{v}, i)} =&\frac{\max \left[ 0, -\varvec{\nabla }_{\textbf{x}_i} U_M \cdot \textbf{v}_i \right] }{C} \bigg [ \\&\quad \delta ^{(3N_a)}\left( \textbf{v}- \textbf{v}' + 2 \, \frac{\textbf{v}' \cdot \varvec{\nabla }_{\textbf{x}} U_{M}}{(\varvec{\nabla }_{\textbf{x}}U_{M})^T \, \textbf{M}^{-1} \, \varvec{\nabla }_{\textbf{x}}U_{M}} \, \textbf{M}^{-1}\,\varvec{\nabla }_{\textbf{x}} U_{M} \right) \\&\quad + \delta ^{(3N_a)}\left( \textbf{v}- \textbf{v}'\right) \bigg ] \\ =&\frac{\max \left[ 0, -\varvec{\nabla }_{\textbf{x}_i} U_M \cdot \textbf{v}_i \right] }{C} \bigg [ \\&\quad \delta ^{(3N_a)}\left( \textbf{v}' - \textbf{v}+ 2 \, \frac{\textbf{v}\cdot \varvec{\nabla }_{\textbf{x}} U_{M}}{(\varvec{\nabla }_{\textbf{x}}U_{M})^T \, \textbf{M}^{-1} \, \varvec{\nabla }_{\textbf{x}}U_{M}} \, \textbf{M}^{-1}\,\varvec{\nabla }_{\textbf{x}} U_{M} \right) \\&\quad + \delta ^{(3N_a)}\left( \textbf{v}' - \textbf{v}\right) \bigg ], \end{aligned} \end{aligned}$$

where the first term applies the force kick, while the second does not. From the necessary condition

9 $$\begin{aligned} 1 = \sum _{k\in \mathcal {N}} \int _{\mathcal {V}^{3N_a}(E_\text {kin})} \textrm{d}^{3N_a}\textbf{v}\, p^M_{(\textbf{v}',j),(\textbf{v},k)}, \end{aligned}$$

it follows that

10 $$\begin{aligned} \begin{aligned} C&= \sum _{k\in \mathcal {N}}\bigg \{\max \left[ 0, -\varvec{\nabla }_{\textbf{x}_k} U_M \cdot \left( \textbf{v}'_k - 2\frac{\textbf{v}' \cdot \varvec{\nabla }_\textbf{x}U_M}{(\varvec{\nabla }_\textbf{x}U_M)^T \, \textbf{M}^{-1}\, \varvec{\nabla }_\textbf{x}U_M} \left( \textbf{M}^{-1}\, \varvec{\nabla }_\textbf{x}U_M\right) _k \right) \right] \\&\quad \quad \quad \,\,\,\,+ \max \left[ 0, -\varvec{\nabla }_{\textbf{x}_k} U_M \cdot \textbf{v}_k'\right] \bigg \}\\&= \sum _{k\in \mathcal {N}}\bigg \{\max \left[ 0, \varvec{\nabla }_{\textbf{x}_k} U_M \cdot \left( \textbf{v}'_k - 2\frac{\textbf{v}' \cdot \varvec{\nabla }_\textbf{x}U_M}{(\varvec{\nabla }_\textbf{x}U_M)^T \, \textbf{M}^{-1}\, \varvec{\nabla }_\textbf{x}U_M} \left( \textbf{M}^{-1}\, \varvec{\nabla }_\textbf{x}U_M\right) _k \right) \right] \\&\quad \quad \quad \,\,\,\,+ \max \left[ 0, \varvec{\nabla }_{\textbf{x}_k} U_M \cdot \textbf{v}_k'\right] \bigg \}. \end{aligned} \end{aligned}$$

The second equation here follows from expressing the equality

11 $$\begin{aligned} \textbf{v}\cdot \varvec{\nabla }_\textbf{x}U_M + \left( \textbf{v}- 2 \frac{\textbf{v}\cdot \varvec{\nabla }_\textbf{x}U_M}{(\varvec{\nabla }_\textbf{x}U_M)^T \, \textbf{M}^{-1} \, \varvec{\nabla }_\textbf{x}U_M} \textbf{M}^{-1} \, \varvec{\nabla }_\textbf{x}U_M \right) \cdot \varvec{\nabla }_\textbf{x}U_M = 0 \end{aligned}$$

as a sum over $k\in \mathcal {N}$ and from grouping the positive and negative terms separately. The normalization factor *C* in $p^M_{(\textbf{v}', j), (\textbf{v}, i)}$ is the same for every possible next active atom *i*. Because of the $\delta ^{(3 N_a)}$-functions in Eq. (8), the normalization factor *C* can also be written in terms of the velocity $\textbf{v}$ after the force kick by replacing $\textbf{v}'\rightarrow \textbf{v}$ in Eq. (10). This yields

12 $$\begin{aligned} \begin{aligned} \mathcal {F}^\text {lift}_M(\textbf{x}, \textbf{v}, i)&= \beta \, \hat{\pi }(\textbf{x}, \textbf{v}, i) \max \left[ 0, -\varvec{\nabla }_{\textbf{x}_i} U_M \cdot \textbf{v}_i\right] \frac{1}{C} \sum _{j\in \mathcal {N}} \bigg \{ \\&\quad \max \left[ 0, \varvec{\nabla }_{\textbf{x}_j} U_M \cdot \left( \textbf{v}_j - 2 \frac{\textbf{v}\cdot \varvec{\nabla }_\textbf{x}U_M}{(\varvec{\nabla }_\textbf{x}U_M)^T \, \textbf{M}^{-1} \, \varvec{\nabla }_\textbf{x}U_M} \left( \textbf{M}^{-1} \, \varvec{\nabla }_\textbf{x}U_M\right) _j\right) \right] \\&\quad + \max \left[ 0, \varvec{\nabla }_{\textbf{x}_j} U_M \cdot \textbf{v}_j \right] \bigg \} \\&= \beta \, \hat{\pi }(\textbf{x}, \textbf{v}, i) \max \left[ 0, -\varvec{\nabla }_{\textbf{x}_i} U_M \cdot \textbf{v}_i\right] \\&= - \mathcal {F}^\text {phys}_M(\textbf{x}, \textbf{v}, i), \end{aligned} \end{aligned}$$

where we used that the velocity $\textbf{v}$ and activity label *i* are uniformly distributed over their sample spaces $\mathcal {V}^{3 N_a}(E_\text {kin})$ and $\mathcal {N}$, respectively. Equation (12) concludes the proof that the presented Newtonian-general scheme for the update of the lifting variables in an event by a general factor potential satisfies the global-balance condition of Eq. (5). The reflective^29^ and forward^40^ event-chain Monte Carlo variants can likewise be generalized to factor potentials depending on an arbitrary number of atoms.

For the Newtonian-pair scheme for distance-dependent pair potentials and identity mass matrix $\textbf{M}=\textbf{I}$, we get

13 $$\begin{aligned} \begin{aligned} p^M_{(\textbf{v}', j), (\textbf{v}, i)}&= {\left\{ \begin{array}{ll} \delta ^{(3N_a)}\left( \textbf{v}- \textbf{v}' + 2 \, \frac{\textbf{v}' \cdot \varvec{\nabla }_{\textbf{x}} U_{M}}{|\varvec{\nabla }_{\textbf{x}}U_{M}|^2} \, \varvec{\nabla }_{\textbf{x}} U_{M} \right) &{} \text {if }i,j\in M\text { and }i \ne j, \\ 0 &{} \text {otherwise,} \end{array}\right. } \\&= {\left\{ \begin{array}{ll} \delta ^{(3N_a)}\left( \textbf{v}' - \textbf{v}+ 2 \, \frac{\textbf{v}^T \cdot \varvec{\nabla }_{\textbf{x}} U_{M}}{|\varvec{\nabla }_{\textbf{x}}U_{M}|^2} \, \varvec{\nabla }_{\textbf{x}} U_{M} \right) &{} \text {if }i,j\in M\text { and }i \ne j, \\ 0 &{} \text {otherwise.} \end{array}\right. } \end{aligned} \end{aligned}$$

With $i,j\in M$ and $i\ne j$, this yields

14 $$\begin{aligned} \begin{aligned} \mathcal {F}^\text {lift}_M(\textbf{x},\textbf{v},i)&= \beta \, \hat{\pi }(\textbf{x},\textbf{v},i) \max \left[ 0, \varvec{\nabla }_{\textbf{x}_j} U_M \cdot \left( \textbf{v}_j - 2 \frac{\textbf{v}\cdot \varvec{\nabla }_\textbf{x}U_M}{|\varvec{\nabla }_{\textbf{x}}U_{M}|^2} \varvec{\nabla }_{\textbf{x}_j} U_M \right) \right] \\&= \beta \, \hat{\pi }(\textbf{x},\textbf{v},i) \max \left[ 0, \varvec{\nabla }_{\textbf{x}_j} U_M \cdot \textbf{v}_j - 2 \frac{\textbf{v}_i \cdot \varvec{\nabla }_{\textbf{x}_i} U_M + \textbf{v}_j \cdot \varvec{\nabla }_{\textbf{x}_j}U_M}{\left| \varvec{\nabla }_{\textbf{x}_i} U_M\right| ^2 + \left| \varvec{\nabla }_{\textbf{x}_j}U_M\right| ^2} \left| \varvec{\nabla }_{\textbf{x}_j} U_M\right| ^2 \right] \\&= \beta \, \hat{\pi }(\textbf{x},\textbf{v},i) \max \left[ 0, -\varvec{\nabla }_{\textbf{x}_i} U_M \cdot \textbf{v}_i \right] \\&= -\mathcal {F}^\text {phys}_M (\textbf{x}, \textbf{v}, i). \end{aligned} \end{aligned}$$

Here, we again used the uniform distributions of $\textbf{v}$ and *i* and the translational invariance $\varvec{\nabla }_{\textbf{x}_i}U_M = -\varvec{\nabla }_{\textbf{x}_j} U_M$. Thus, also the Newtonian-pair scheme satisfies the global-balance condition of Eq. (5).

### Fibonacci vectors

The cell-veto algorithm was previously only used for the straight variant of event-chain Monte Carlo^23,24,27^. Its finite set of possible velocities $\mathcal {D}$ of the single active atom *a* allows one to calculate a Walker table for every velocity $\textbf{v}_a\in \mathcal {D}$. For instance, for factor pair potentials between the active atom *a* and another atom *b* at positions $\textbf{x}_a$ and $\textbf{x}_b$, $U_M(\textbf{x}) = U_M(\textbf{x}_a, \textbf{x}_b)$, every cell bound $q_M^\text {cell}(\mathcal {C}_a, \mathcal {C}_b, \textbf{v}_a)$ for the pair of cells $\mathcal {C}_a$ and $\mathcal {C}_b$ in the Walker table may be written as

15 $$\begin{aligned} q_M^\text {cell}(\mathcal {C}_a, \mathcal {C}_b, \textbf{v}_a) = \max _{\textbf{x}_a \in \mathcal {C}_a, \textbf{x}_b \in \mathcal {C}_b} \beta \max \left[ 0, \varvec{\nabla }_{\textbf{x}_a} U_M (\textbf{x}_a, \textbf{x}_b) \cdot \textbf{v}_a\right] . \end{aligned}$$

In principle, a Walker table must be precomputed for every velocity $\textbf{v}_a\in \mathcal {D}$ and for every possible cell $\mathcal {C}_a$ of the active atom. Symmetries, such as a translational invariance of the factor potential, may heavily reduce the necessary number of Walker tables^23^. During an actual simulation with the straight event-chain Monte Carlo algorithm, the relevant Walker table is determined by the cell of the currently active atom and its velocity. The generalization of Eq. (15) to more complex factors is straightforward. For example, in order to treat the Coulomb interaction between two water molecules in the SPC/Fw water model, the position and orientation of the water molecule containing the active atom may be varied in $\mathcal {C}_a$, while the position and orientation of the other molecule is varied in $\mathcal {C}_b$. From a practical point of view, the cell bounds $q_M^\text {cell}(\mathcal {C}_a, \mathcal {C}_b)$ are usually not computed exactly but rather approximated, e.g., by considering a finite set of positions $\textbf{x}_a\in \mathcal {C}_a$ and $\textbf{x}_{b}\in \mathcal {C}_b$, and including a corrective multiplicative prefactor^24^. As long as the approximated cell bound satisfies $\tilde{q}^\text {cell}_M (\mathcal {C}_a, \mathcal {C}_b, \textbf{v}_a) \ge q^\text {cell}_M(\mathcal {C}_a,\mathcal {C}_b, \textbf{v}_a)$, a Poisson thinning procedure corrects *any* overestimate^26^. The quality of $\tilde{q}^\text {cell}_M (\mathcal {C}_a, \mathcal {C}_b, \textbf{v}_a)$, however, does influence the performance. Higher cell bounds in the cell-veto algorithm yield more events per unit distance that have to be confirmed by computing the actual event rate.

The inherent discretization of the continuous-position space in Eq. (15) can be translated to a continuous velocity space, as it appears, e.g., in generalized Newtonian event-chain Monte Carlo. Consider a finite number of unit vectors $\hat{\textbf{v}}_d$ on the two-dimensional unit sphere, and let $\mathcal {V}_d$ be the associated Voronoi cells under some distance function. We can then formally compute the cell bounds for every Voronoi cell as

16 $$\begin{aligned} q_M^\text {cell}(\mathcal {C}_a, \mathcal {C}_b, \mathcal {V}_a) = \max _{\textbf{x}_a\in \mathcal {C}_a, \textbf{x}_b \in \mathcal {C}_b,\textbf{v}_a\in \mathcal {V}_a} \beta \max \left[ 0, \varvec{\nabla }_{\textbf{x}_a} U_M (\textbf{x}_a, \textbf{x}_b) \cdot \textbf{v}_a\right] . \end{aligned}$$

The results are used to precompute Walker tables for every Voronoi cell $\mathcal {V}_a$ and for every cell $\mathcal {C}_a$, where symmetries may again heavily reduce the number of actually necessary tables. During the generalized Newtonian event-chain Monte Carlo simulation, the relevant Walker table is determined by finding the cell of the currently active atom and the Voronoi cell of its normalized velocity $\textbf{v}_a / |\textbf{v}_a|$. We can then correct all cell bounds in the relevant Walker table to the actual speed of the active atom by multiplying them with $|\textbf{v}_a|$.

In this paper, as proof of concept, we map a generalized Fibonacci lattice onto a two-dimensional unit sphere by the Lambert cylindrical equal-area projection to generate *D* unit vectors $\hat{\textbf{v}}_d^\text {fib}$:

17 $$\begin{aligned} \hat{\textbf{v}}_d^\text {fib} = \left( \cos \phi _d \sin \theta _d, \sin \phi _d\sin \theta _d, \cos \theta _d \right) ^T, \end{aligned}$$

where $$0\le d < D$$ and

18 $$\begin{aligned} \begin{aligned} \phi _d&= \frac{2\pi d}{\varphi },\\ \theta _d&= \arccos \left( 1 - \frac{2 (d + \varepsilon )}{D - 1 + 2\varepsilon }\right) , \end{aligned} \end{aligned}$$

with the golden ratio $\varphi = (1 + \sqrt{5})/2$^63–65^. The empirical choice of the parameter $\varepsilon ={0.36}$ optimizes the average nearest-neighbor distance of the Fibonacci vectors $\hat{\textbf{v}}_d$. As a distance function to construct the Voronoi cells $\mathcal {V}_d$, we use the quick-to-evaluate cosine distance. Equations (17) and (18) efficiently generate an arbitrary number of Fibonacci vectors with a nearly uniform distribution. However, there is no efficient inverse mapping from a general velocity $\textbf{v}_a$ to the closest Fibonacci vector $\hat{\textbf{v}}_d$. This is not a problem in this paper because we choose $D=10$ small. Then, $\hat{\textbf{v}}_d$ can be found by brute force. If considerably larger values of *D* become necessary, other point configurations on the two-dimensional unit sphere may be considered^65,66^. For the uniform SPC/Fw water systems in this paper, the Walker tables do not strongly depend on the respective Fibonacci vector. The proper discretization of velocity space will, however, impact the performance of Newtonian event-chain Monte Carlo in nonuniform systems.

### SPC/Fw water model

This paper benchmarks the event-chain Monte Carlo algorithm, molecular dynamics, and the Metropolis algorithm for molecular simulations of the atomistic flexible simple point-charge SPC/FW water model^34^. It defines a water molecule by three charged interaction sites that represent the oxygen and hydrogen atoms. We treat the canonical ensemble in a cubic box with periodic boundary conditions, i.e., *N* water molecules with $N_a=3N$ atoms in a periodically repeated cubic box of side length *L* at a given temperature $T\sim {300}\,{\hbox {K}}$. Within any water molecule $i\in \{1,\ldots ,N\}$, the intramolecular interactions of the SPC/Fw model consist of two harmonic bond potentials $U_{\text {bond}}^{i,1}$ and $U_{\text {bond}}^{i,2}$ that lead to fluctuations of the O–H bond lengths around their equilibrium length. Likewise, a harmonic bending potential $U^{i}_\text {bend}$ yields a fluctuation of the H–O–H opening angle around an equilibrium value. The intermolecular interactions between two different water molecules *i* and *j* consist of a Lennard-Jones potential $U^{ij}_\text {LJ}$ between the two oxygen atoms, and a Coulomb potential $U^{ij}_\text {C}$ between all nine pairs of charged atoms. The intermolecular potentials explicitly include the interactions between all periodic images of the two involved water molecules. The total potential *U* of an all-atom configuration $\textbf{x}$ is a sum of these factor potentials:

19 $$\begin{aligned} U(\textbf{x}) = \sum _{i=1}^{N} \left[ U_{\text {bond}}^{i,1}(\textbf{x}) + U_{\text {bond}}^{i,2}(\textbf{x}) + U_{\text {bend}}^i(\textbf{x}) \right] + \sum _{i=1}^N \sum _{j=1}^{i-1} \left[ U_\text {LJ}^{ij}(\textbf{x}) + U_\text {C}^{ij}(\textbf{x}) \right] . \end{aligned}$$

The bond and Lennard-Jones factor potentials depend on two atomic positions; the bending and Coulomb factor potentials depend on three and six atomic positions, respectively (see also Ref.^23^, Section V A). The empirical parameters of the different potentials of the SPC/Fw water model are as in Ref.^34^.

### Simulation protocols—molecular dynamics

**Figure 5:**
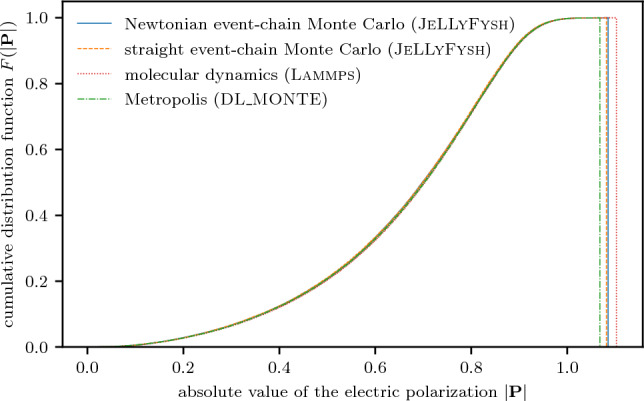
Validation for the SPC/Fw water model. Cumulative distribution function of the absolute value of the electric polarization of $N=2$ SPC/Fw water molecules in a cubic box of side length $L={20}$ Å.

We use the feature release from February 8, 2023, of lammps^8,67^ for the molecular-dynamics simulations of the SPC/Fw water model in this paper. We employ a spherical cut-off for the Lennard-Jones potential. The Coulomb potential is treated by a particle–particle particle–mesh solver ^12^. Unless explicitly specified otherwise (as, e.g., in Fig. 4b), the solver uses a target accuracy of $10^{-6}$. Coulomb interactions are partly treated in discretized reciprocal space, whereby the grid size is chosen to meet the target accuracy based on analytic error estimates obtained from a specific global charge distribution^15,48,49^. Thermostatting is achieved by integrating the Nosé–Hoover-chain equations of motion for the canonical ensemble^68^ with a time-reversible measure-preserving Verlet integrator^69^. The time step is ${1}\,\hbox {fs}$, and the temperature is relaxed in a timespan of roughly 300 time steps. This simulation protocol for molecular dynamics in lammps is validated by comparing numerical results for $N=2$ SPC/Fw water molecules of all sampling methods of this paper (see Fig. 5).

### Simulation protocols—Metropolis algorithm

We use version 2.07 of the dl_monte software package^47,70,71^ to sample the canonical ensemble of the SPC/Fw water model with the reversible Metropolis algorithm^37^, and minimally amended the software to output the electric polarization. We employ a spherical cut-off for the Lennard-Jones potential. The Coulomb interaction is treated by an Ewald summation^72^ with a target-accuracy tolerance of $10^{-6}$. The simulations with the non-reversible event-chain Monte Carlo algorithm in this paper move a single atom at a time. For a direct comparison to the dynamics of the reversible Metropolis algorithm (see Fig. 3c), each trial proposes a new position of a single random atom in a proposal cube around its original position. The new position is accepted with the usual Metropolis criterion based on the change of energy. The size of the proposal cube is separately adapted for the hydrogen and oxygen atoms during the simulation to obtain a target acceptance rate of $37\%$. This simulation protocol for the Metropolis algorithm in dl_monte is validated by comparing numerical results for $N=2$ SPC/Fw water molecules of all sampling methods of this paper (see Fig. 5).

### Simulation protocols—event-chain Monte Carlo

We develop version 2.0 of jellyfysh^24,56^ for the event-chain Monte Carlo simulations of the SPC/Fw water model in this paper. In addition to the straight variant implemented in versions $< 2.0$, version 2.0 implements the generalized Newtonian event-chain Monte Carlo variant. We use the factorization in Eq. (19). In particular, we group all the charge–charge Coulomb interactions between two water molecules into a single factor. Theory then predicts an optimal logarithmic increase in the number of events from these molecular Coulomb factors^23^ that is numerically confirmed in Fig. 3b.

Straight event-chain Monte Carlo (used in Fig. 3c) periodically aligns the unit velocity $|\textbf{v}_a|= 1$ of the active atom with the coordinate axes. We change the velocity after a chain time of $\tau _\text {chain}=0.2N$^43^. In an event triggered by a factor *M*, the velocity is transferred to another atom which belongs to *M*. For factors with more than two atoms, the next active atom is chosen according to the ratio lifting scheme^23,39^.

Generalized Newtonian event-chain Monte Carlo assigns a velocity label to every atom, but only moves a single one at any time. The velocities are initialized so that the average speed of the active atom during the simulation is approximately one: $\langle |\textbf{v}_a| \rangle \approx 1$. This is possible because the velocities have no kinematic meaning as they have in molecular dynamics. The physical temperature only rescales the event rates [see Eq. (1)]. The velocities are resampled in large time intervals of $\tau _\text {chain}={10000}{N}$. This is because Newtonian event-chain Monte Carlo rotates dipoles most efficiently in the limit $\tau _\text {chain}\rightarrow \infty$, while a finite value of $\tau _\text {chain}$ ensures irreducibility^41,57^. Although our generalized Newtonian event-chain Monte Carlo can consider a general mass matrix of the atoms in its events, we set the masses of all atoms to be equal. Then, the events of factors *M* of two atoms can be treated with the deterministic Newtonian-pair scheme in which the two involved velocities and the active atom always change. For factors *M* of more than two atoms, we apply the stochastic Newtonian-general scheme that corresponds to a generalized ratio lifting scheme.

The events of the bond factors are computed exactly [see Eq. (2)], while the bending factors are treated with a piecewise-linear bounding potential followed by a Poisson thinning procedure (see Ref.^24^, Section 4.4.5). The cell-veto algorithm^27^ bundles most molecular Coulomb factors and relies on a cell-occupancy system that tracks the molecular barycenters (we generally adapt the setup described in Ref.^24^, Section 5.3.4). Very close molecule pairs or surplus molecules in the cell-occupancy system are separately treated with a piecewise-linear bounding potential. The cell bounds for 10 different Fibonacci vectors are estimated by varying the position of an atom in one cell, and the position of a dipole in the other cell. Here, the dipole is aligned with the direction of the gradient of the charge–dipole Coulomb interaction. The maximum event rate multiplied by an empirical prefactor yields the cell bound. During the simulation, the cell bounds in the Walker table are rescaled to the actual speed and charge of the active atom. Events from the cell-veto algorithm are confirmed with a Poisson thinning procedure that compares the actual event rate of the Coulomb interaction between two molecules with the sampled cell bound. To compute the real event rate, we compute the gradients of the Coulomb potential between the nine pairs of charged atoms [see also Eq. (1)] with an Ewald summation^72^ that is tuned to machine precision without any assumption on the global charge distribution. We also use the cell-veto algorithm to treat most Lennard-Jones factors, although we cut off the interaction beyond the closest images for consistency with the other sampling algorithms. We carefully checked that an alternative spherical cut-off does not change the results of this paper. The cell-veto algorithm for the Lennard-Jones interaction relies on a cell-occupancy system that tracks oxygen atoms. Very close or surplus oxygen pairs are treated directly [see Eq. (2)]. The cell bounds for 10 different Fibonacci vectors are estimated by varying the oxygen positions evenly in the two cells, and again including an empirical prefactor. The parameters of the cell-occupancy systems, as the cell sizes and the number of nearby cells that are excluded from the cell-veto algorithm, are tuned to minimize computer time. As a rule of thumb, the largest possible cells that do not yield any surplus water molecules, and two excluded layers are found to be a good choice.

These simulation protocols for straight and generalized Newtonian event-chain Monte Carlo in jellyfysh are validated by comparing numerical results for $N=2$ SPC/Fw water molecules of all sampling methods of this paper (see Fig. 5).

### Simulation protocols—creation of initial configurations

We create initial configurations in the range from $N=64$ to $N=2744$ SPC/Fw water molecules with hydrogen mass $m_{{H}} = {1.0079}\,{\hbox {Da}}$ and oxygen mass $m_{{O}} = {15.9994001}\,{\hbox {Da}}$ at a density of $\rho ={0.97}\,\hbox {g\,cm}^{-3}$ using the software package playmol^73^ (commit 67eb56c from 26 November 2019). Initial configurations are equilibrated using a molecular-dynamics simulation of lammps in the isothermal–isobaric ensemble at pressure $P={1}\,\hbox {atm}$. Similar to thermostatting, barostatting is achieved by integrating the appropriate Nosé–Hoover-chain equations of motion with a Verlet integrator^68,69^ with a time step of ${1}\,\hbox {fs}$. This results in initial configurations for the simulations in the canonical ensemble with the different sampling algorithms at slightly different densities for different *N*. We confirmed that these small density variations do not influence the results of this paper.

### Analysis of electric polarization

We sample the electric polarization $\textbf{P}$ (or the total electric dipole moment $\textbf{P}=\sum _{i=1}^N \textbf{p}_i$, where $\textbf{p}_i$ is the molecular dipole moment of the water molecule *i*) of the SPC/Fw water system with the different sampling algorithms. The generated “time”-series $\textbf{P}(t)$ (where the “time” *t* only has a physical meaning in molecular dynamics) yields the normalized autocorrelation function

20 $$\begin{aligned} \Phi _\textbf{P}(\tau ) = \frac{\langle \textbf{P}(t)\cdot \textbf{P}(t + \tau )\rangle _t}{\langle \textbf{P}(t)\cdot \textbf{P}(t)\rangle _t}, \end{aligned}$$

where we explicitly assume that $\langle \textbf{P} \rangle = 0$ for $t\rightarrow \infty$. The first part of each trajectory in the canonical ensemble is not considered in the equilibrium average. We observe an exponential decay in all sampling algorithms and fit an exponential $\sim \exp (-\tau /\tau _\Phi )$ to extract the time constant $\tau _\Phi$ and its standard error (see also Ref.^34^). We naïvely parallelize the Metropolis and event-chain Monte Carlo algorithms by running 20 to 50 ﻿independent simulations (where the number of runs grows with *N*). For every $\tau$, we then compute the median autocorrelation function $\Phi _\textbf{P}(\tau )$ and estimate its error with the bootstrap method. These errors are then considered in the least-squares fit.

The units of the autocorrelation time $\tau _\Phi$ for the different sampling algorithms are physical time for molecular dynamics, Monte-Carlo trials for the Metropolis algorithm, and continuous Monte-Carlo time for the event-chain Monte Carlo algorithms. As a first measure of the efficiency of the different dynamics, we compare the autocorrelation distances $d_\Phi$ (see Fig. 3c). It gives the average cumulative distance moved by the atoms in each of the sampling schemes. Since only a single atom moves in our implementation of the event-chain Monte Carlo algorithm, it follows that $d_\Phi ^\text {ECMC}=\langle |\textbf{v}_a|\rangle ^\text {ECMC} \, \tau _\Phi ^\text {ECMC}$, where $\langle |\textbf{v}_a|\rangle ^\text {ECMC}$ is the average speed of the active atom during the simulation. For molecular dynamics, we can compute the average speed of the hydrogen and oxygen atoms $t\in \{{H}, {O}\}$ with the Maxwell–Boltzmann distribution:

21 $$\begin{aligned} \langle \left| \textbf{v}_t \right| \rangle ^\text {MD} = \sqrt{\frac{8 k_\text{B} T}{\pi m_t}}, \end{aligned}$$

which yields $d_\Phi ^\text {MD}=N (2 \langle |\textbf{v}_{{H}}| \rangle ^\text {MD} + \langle | \textbf{v}_{{O}} | \rangle ^\text {MD}) \, \tau _\Phi ^\text {MD}$. In the Metropolis algorithm, each trial samples a random displacement vector $\textbf{d}_t=[{{\,\textrm{ran}\,}}(-\delta _t, \delta _t), {{\,\textrm{ran}\,}}(-\delta _t, \delta _t), {{\,\textrm{ran}\,}}(-\delta _t, \delta _t)]^T$ for a random atom, where $\delta _t$ is tuned independently for the oxygen and hydrogen atoms $t\in \{ {H}, {O} \}$ to accept the displacement with probability $p\approx 0.37$. We can compute the average length $\langle |\textbf{d}_t|\rangle ^\text {Met}$ of the sampled displacement vector as

22 $$\begin{aligned} \langle |\textbf{d}_t|\rangle ^\text {Met} = \frac{1}{8\delta _t^3}\int _{-\delta _t}^{\delta _t}\textrm{d}x\, \int _{-\delta _t}^{\delta _t}\textrm{d}y\, \int _{-\delta _t}^{\delta _t}\textrm{d}z\, \sqrt{x^2 + y^2 + z^2} \approx {0.961}\,\delta _t. \end{aligned}$$

Neglecting correlations between rejection probability and the displacement for this first measure of efficiency, we get $d_\Phi ^\text {Met}=p\,(2\langle |\textbf{d}_{{H}}|\rangle ^\text {Met}/3 + \langle |\textbf{d}_{{O}}|\rangle ^\text {Met}/3)\,\tau _\Phi ^\text {Met}$.

The computer autocorrelation time of our generalized Newtonian event-chain Monte Carlo algorithm (see Fig. 4b) is obtained by combining the autocorrelation distance (see Fig. 3c), the event rates (see Fig. 3b), and the computer time per event in jellyfysh (see Fig. 3a). Similarly, we measure the computer time per time step of molecular dynamics in lammps for different target accuracies of its particle–particle particle–mesh solver (see Fig. 4a). This is then combined with the autocorrelation time in the number of time steps.

## Data Availability

The jellyfysh software is made available under the GNU GPLv3 license at https://github.com/jellyfysh. All configuration files for the simulations of this paper are part of jellyfysh.
